# An Inhibitory Motif on the 5’UTR of Several Rotavirus Genome Segments Affects Protein Expression and Reverse Genetics Strategies

**DOI:** 10.1371/journal.pone.0166719

**Published:** 2016-11-15

**Authors:** Giuditta De Lorenzo, Marija Drikic, Guido Papa, Catherine Eichwald, Oscar R. Burrone, Francesca Arnoldi

**Affiliations:** 1 International Centre for Genetic Engineering and Biotechnology, Trieste, Italy; 2 Department of Medicine, Surgery and Health Sciences, University of Trieste, Trieste, Italy; 3 Institute of Virology, University of Zurich, Zurich, Switzerland; Kliniken der Stadt Köln gGmbH, GERMANY

## Abstract

Rotavirus genome consists of eleven segments of dsRNA, each encoding one single protein. Viral mRNAs contain an open reading frame (ORF) flanked by relatively short untranslated regions (UTRs), whose role in the viral cycle remains elusive. Here we investigated the role of 5’UTRs in T7 polymerase-driven cDNAs expression in uninfected cells. The 5’UTRs of eight genome segments (gs3, gs5-6, gs7-11) of the simian SA11 strain showed a strong inhibitory effect on the expression of viral proteins. Decreased protein expression was due to both compromised transcription and translation and was independent of the ORF and the 3’UTR sequences. Analysis of several mutants of the 21-nucleotide long 5’UTR of gs 11 defined an inhibitory motif (IM) represented by its primary sequence rather than its secondary structure. IM was mapped to the 5’ terminal 6-nucleotide long pyrimidine-rich tract 5’-GGY(U/A)UY-3’. The 5’ terminal position within the mRNA was shown to be essentially required, as inhibitory activity was lost when IM was moved to an internal position. We identified two mutations (insertion of a G upstream the 5’UTR and the U to A mutation of the fifth nucleotide of IM) that render IM non-functional and increase the transcription and translation rate to levels that could considerably improve the efficiency of virus helper-free reverse genetics strategies.

## Introduction

Group A rotaviruses (*Reoviridae* family) are the major cause of gastroenteritis in infants and young children worldwide and infect many other animal species [1,2]. Rotavirus genome consists of eleven segments of linear double-stranded RNA (dsRNA). The complementary strands of each genome segment (gs) are base paired from end to end [3]. The plus strand of the duplex contains a 5’-terminal m^7^GpppG^m^ cap and both strands lack poly(A) tails [3,4]. The viral RNA-dependent RNA polymerase VP1 transcribes plus-stranded RNAs that act both as messengers for the synthesis of viral proteins and as templates for the synthesis of new gs. Each viral messenger contains one single open reading frame (ORF) flanked by rather short untranslated regions (UTRs) with the exception of gs11 that in some strains encodes a second protein through an additional ORF [1,2]. UTRs are believed to play a role in genome replication, genome packaging, and in the regulation of gene expression, as a consequence of either their primary nucleotidic sequences or their secondary structures. Although UTRs are not completely conserved among the different gs (and often differ in their length), terminal consensus sequences are common to all eleven gs: the 5’-terminal consensus 5’-GGC(A/U)_7_−3’ and the 3’-terminal consensus 5’-U(G/U)_3_(A/G)CC-3’. It has been shown that the 7-nucleotide long 3’-terminal consensus in the plus strand represents the minimal promoter for the minus strand synthesis of the genome replication reaction (catalyzed also by the viral polymerase VP1), with the terminal cytidine residues (CC) essentially required. Additional sequences at the 5’ end and upstream of the 3’-terminal consensus enhance the process without being essential [5,6]. Based on secondary structure predictions these additional sequences were proposed to form a panhandle structure from which the 3’-terminal consensus protrudes as a single-stranded tail [7], which is possibly bound by VP1 during the replication reaction.

Rotavirus studies have always been affected by the lack of a reverse genetics technique. Helper virus-free reverse genetics methods based on the introduction of mRNAs into the cytosol were already successful with other viruses belonging to the same family of rotavirus (orbivirus or orthoreovirus). In particular, in the case of orthoreovirus, mRNAs were generated by T7 polymerase-driven transcription of transfected cDNAs [8,9], while in the case of orbivirus co-transfection of all ten full-length plus-stranded transcripts was successful [10]. These achievements indicate that the mRNAs of the high-number segmented dsRNA viruses are infectious and that also for rotavirus the establishment of a helper virus-independent reverse genetics system is theoretically achievable. Rotavirus, however, appears to be recalcitrant to these techniques. A few successful strategies have been reported, which unfortunately cannot be extended to any rotavirus genome segment and are all helper virus-driven procedures [11–15]. In all these cases, the strategies are based on transfection of viral cDNAs cloned under the control of the T7 promoter and flanked at the 3’ end by a modified hepatitis delta virus (HDV) ribozyme; this system ensures transcription of a messenger that perfectly mimics the authentic ends of rotavirus mRNAs but is limited by the inefficiency in rescuing recombinant viruses from the background of progeny helper viruses, thus requiring strong selection methods.

The attempts to obtain a recombinant rotavirus after co-transfection of the complete set of full-length rotavirus transcripts independently of the use of a helper virus have failed [16]. In this work we show that the low expression levels of most single proteins expressed *in vivo* are the consequence of a strong inhibitory motif (IM) present on the 5’UTRs of eight genome segments (3, 5–6, 7–11) of several RV strains. This could explain, at least in part, the failure of the systems so far tested.

## Materials and Methods

### Cells and viruses

MA104 (embryonic African green monkey kidney) and HeLa (human cervical epithelial adenocarcinoma) cells were grown in Dulbecco’s Modified Eagle’s Medium (DMEM) (Life Technologies) containing 10% Fetal Bovine Serum (FBS) (Life Technologies) and 50 μg/ml gentamycin (Biochrom AG). For infection experiments, the following viruses were used: rotavirus SA11 4F strain (G3P6[1]); T7-recombinant vaccinia virus (strain vTF7.3) [17] that delivers the T7 RNA polymerase necessary for the production of viral-like messengers; vT7-NE virus, an IPTG-inducible recombinant vaccinia virus driving expression of both the T7 RNA polymerase and a gs11-like construct whose ORF encodes the fusion protein NSP5-EGFP. The virus vT7-NE was produced as previously described [18].

### Transient transfections

Transient transfections were performed on confluent monolayers of MA104 or HeLa cells grown in six-well plates (Falcon). MA104 and HeLa cells were infected with vTF7.3 at an MOI of 10 [17,19]. At 1 hour post-infection (h.p.i.) cells were transfected with 2 μg/well of plasmid DNA using Lipofectamine 3000 (Life Technologies) according to the manufacturer’s instructions. At 16 h.p.i. cells were lysed with 100 μl of reducing SDS buffer (125 mM Tris-HCl pH 6.8, 6% SDS, 40% glycerol, 5% β-mercaptoethanol, 0.04% bromophenol blue) and then sonicated with a VialTweeter (Hielscher Ultrasonics GmbH) for 1 min (10 W, pulse 0.5 sec) to disrupt DNA.

For the experiments of *in vivo* translation of pre-synthesized mRNAs, MA104 cells were electroporated with 3 μg of polyadenylated mRNAs using the Amaxa/Lonza Nucleofector Technology (U-020 program; V solution); at 4 h post-transfection cells were lysed and treated as described above.

For combined transfection and rotavirus infections, confluent monolayers of MA104 cells in twelve-well plates were infected with vT7-NE virus (MOI 100) and 1 h later were induced with 1 mM IPTG and infected with SA11 (MOI 5). At 9 h post-rotavirus infection cells were lysed as described above.

### Western blot analyses

Samples were separated in 12% SDS-PAGE using Precision Plus Protein Standards molecular mass markers (Bio-Rad) and then transferred to polyvinylidene difluoride membranes (Millipore, IPVH00010). Membranes were blocked in 5% milk/PBS and then incubated with the appropriate antibody. Primary antibodies: anti-NSP5 guinea pig serum, anti-VP2 guinea pig serum, anti-NSP2 guinea pig serum, anti-EGFP mouse monoclonal serum (Santa Cruz Biotechnology), anti-α-tubulin mouse monoclonal antibody (Calbiochem), anti-α-actinin rabbit polyclonal antibody (H-300, Santa Cruz Biotechnology). Guinea pig sera for NSP5, NSP2 and VP2 were previously described [20–22]. Secondary antibodies: HRP-conjugated goat anti-guinea pig (Jackson ImmunoResearch), goat anti-mouse (Jackson ImmunoResearch), goat anti-rabbit (Thermo Scientific Pierce). Signals were detected by using the enhanced chemiluminescence system (Pierce ECL Western Blotting Substrate, Thermo Scientific).

### *In vitro* transcription

Plasmids (1 μg) linearized with SacII restriction enzyme (NEB) were transcribed by the mMessage mMachine T7 Ultra kit (Life Technologies Ambion) according to the manufacturer’s instructions. Reaction mixtures were incubated at 37°C for 2 h and then treated with TURBO DNase. Polyadenylation of the mRNAs used in nucleofection experiments was performed after transcription following the poly(A) tailing procedure of the mMESSAGE mMACHINE kit. *In vitro* transcripts were purified by the MEGAclear kit (Life Technologies Ambion) and quantified using the Qubit RNA Assay Kit with the Qubit 2.0 Fluorometer (Thermo Fisher Scientific).

### *In vitro* transcription/translation

*In vitro* coupled transcription and translation of plasmids was performed with the 1-Step Human Coupled IVT Kit–DNA (Thermo Scientific) following the manufacturer’s instructions. 1 μg of circular DNA or 1 μg of *in vitro* synthesized mRNAs were used as templates. The reaction mixtures were incubated for 6 h at 30°C and were stopped with 8 μl loading buffer for PAGE and Western Blot analysis.

### Quantitative real-time PCR

For quantification of transcripts, 1 μg of total RNA from each sample was retro-transcribed using random hexamers (IDT), and the resulting product used as a template for quantitative PCR (qPCR) in the SsoFast EvaGreen Supermix (BioRad) and with specific primer sets for EGFP (forward, 5’-TCAAGGAGGACGGCAACATC-3’; reverse, 5’-TTGTGGCGGATCTTGAAGTTC-3’). For all amplification reactions, a 7000 ABI Prism instrument (Life Technologies Applied Biosystems) was used. Relative gene expression was calculated according to the formula 2^**^-Ct**^.

### RNA stability assay

The RNA stability assay was performed using the Click-iT® Nascent RNA Capture Kit (Life Technologies). 3.5 h after DNA transfection, cells were treated with 10 μg/mL cycloheximide (Sigma) and 10 μM pactamycin (Sigma). At 4 h post transfection, the medium was replaced with DMEM containing 0.2 mM 5-ethynyl uridine (EU). 5 μg/mL actynomycin D (Sigma) was added 90 min post treatment with EU. Samples were collected 1.5, 2 and 4 h post EU incubation and total RNA was extracted with the RNeasy Plus Mini kit (Qiagen). Labelled RNAs were conjugated with an azide-modified biotin through a copper catalyzed click reaction. Streptavidin magnetic beads were used to capture labelled RNAs, which were then used as templates for reverse transcriptase-mediated cDNA synthesis for subsequent analysis using qPCR.

### Plasmid constructs

The SA11 5’ UTRs were designed for cloning according to the NCBI reference sequences NC_011500-NC_011510 (RVA/Simian-tc/ZAF/SA11-H96/1958/G3P5B[2]). The plasmid pVAX-T7-segment11-ribo-T7stop containing the sequence coding for rotavirus segment 11 was obtained as described previously [23]. For segments 2 and 8 the same general scheme of construct with T7 promoter and ribozyme and T7 terminator (T7-segment-ribozyme-T7term) was cloned in pUC19 vector (Life Technologies), between BamHI and SacII sites. The various versions of genetic constructs containing rotavirus genome segments either full-length or lacking one or both UTRs were constructed by PCR amplification. The primers used to this end are listed in S1 Table. Point mutations and/or deletions or insertions were obtained by site-specific mutagenesis using the QuikChange II XL Site-Directed Mutagenesis Kit (Agilent). The primers used to this purpose are listed in S2 Table. The EGFP coding sequence was cloned in the pVAX-T7-segment11-ribo-T7stop vector [23] replacing segment 11 with the EGFP ORF. The different UTRs from rotavirus gs were introduced upstream and/or downstream of EGFP by PCR amplifications (primers in S1 Table). The XhoI restriction site was introduced by site-specific mutagenesis in the EGFP ORF (primers in S2 Table). The 5’ UTRs of the remaining rotavirus gs were cloned upstream of EGFP by insertion of the oligonucleotides listed in S3 Table between the KpnI and XhoI sites.

The constructs pcDNA3-NSP3 and pcDNA3-SV5-NSP3 were engineered by cloning the SA11 NSP3 ORF between KpnI and EcoRI restriction sites and the N-terminal SV5 tag [24] between HindIII and KpnI restriction sites of the pcDNA3 vector.

## Results

### The 5’UTR of gs11 mRNA downregulates NSP5 expression

**Fig 1:**
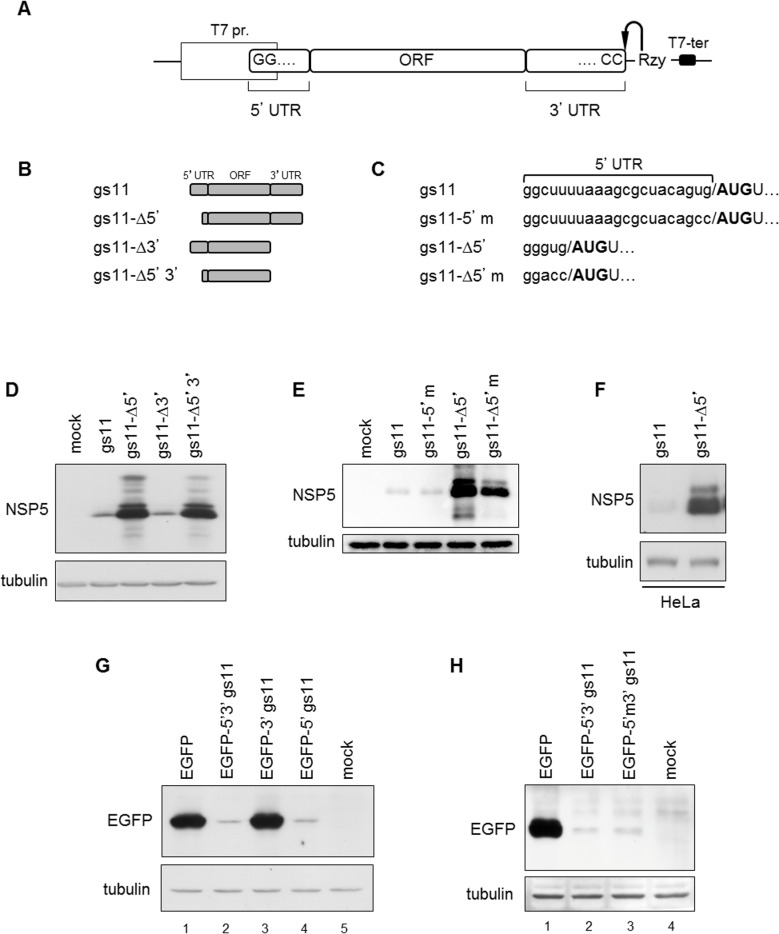
The 5’UTR of gs11 downregulates NSP5 expression. A) Schematic representation of the genetic constructs used. B) Scheme of the different gs11 constructs with one or both UTRs deleted. C) 5’UTR sequences of wild-type gs11 and 5’ deletion mutant (gs11-Δ5’) and of the corresponding variants with mutations on the Kozak sequence (gs11-5’m and gs11-Δ5’m); start codon in bold. D-F) Anti-NSP5 WB of MA104 cells (D, E) or HeLa cells (F) transfected with the indicated constructs. G, H) Anti-EGFP WB of MA104 cells transfected with constructs containing the EGFP ORF with or without gs11 5’ and 3’ UTRs (G) and with a construct (EGFP-5’m3’ gs11) with mutated Kozak sequence (H). Tubulin used as loading control.

Previous results have shown that rotavirus NSP5 is poorly expressed in cells transfected with plasmids containing the full-length gs11 cDNA, but not with plasmids encoding only the NSP5 open reading frame (ORF). In order to investigate whether this depended on the 5’ or 3’ untranslated regions (5’UTR, 3’UTR), we engineered plasmid constructs containing either the full-length sequence of gs11 cDNA or with one (gs11-Δ5'; gs11-Δ3') or both UTRs deleted (gs11-Δ5'3') under the control of the T7 promoter. All plasmid constructs contained the antigenomic hepatitis delta virus (HDV) ribozyme sequence followed by a T7 terminator, to mediate precise transcript processing at the 3' terminal CC dinucleotide. In all constructs, a dinucleotide GG at the 5’ end of the transcribed mRNA was maintained, as it is present in the (+)RNAs of all groups of rotavirus. This dinucleotide corresponds also to the terminal part of the T7 promoter. A schematic representation of the constructs and the sequences at the 5’ ends are shown in Fig 1A–1C.

The different constructs were tested by transfection into MA104 cells, previously infected with a T7 RNA polymerase-encoding recombinant vaccinia virus (vTF7.3), and analyzed by Western blot (WB). As shown in Fig 1D, deletion of both UTRs (gs11-Δ5’3’) or only 16 out of the 21 nucleotides of the 5'UTR (gs11-Δ5') produced a conspicuous increase in protein expression. This increase was not dependent on the nucleotide sequence context of the initiation codon. In fact, the ideal context for efficient translation of vertebrate mRNAs is the RCC**AUG**G sequence (R represents purine; start codon in bold), which is known as the "Kozak sequence" [25]. Nucleotides at positions -3 and +4 are the most important and they are the same in the mutant gs11-Δ5' (GUG**AUG**U) and in the wild-type gs11 construct (GUG**AUG**U). In addition, mutation of the translation initiation viral sequence into an optimized Kozak sequence both in wild-type gs11 (GUG**AUG**U into GCC**AUG**U, gs11-5’m) and in gs11 with the viral 5’UTR deleted (GUG**AUG**U into ACC**AUG**U, gs11-Δ5'm) did not improve expression (Fig 1E).

The negative regulatory effect on expression of gs11 5'UTR was observed also in HeLa cells infected with vTF7.3 (Fig 1F).

To test whether this effect was also dependent on the viral coding region, we obtained constructs in which the gs11 ORF was replaced by an ORF coding for EGFP. As in the case of the viral protein, reduced EGFP expression was only observed with constructs containing both 5' and 3’ gs11 UTRs or only the 5’UTR (Fig 1G). Also in this case an optimized Kozak sequence at the AUG initiation codon (EGFP-5’m3’ gs11) did not alter EGFP expression (Fig 1H, lane 3).

### The 5’UTRs of gs2 and gs8

**Fig 2:**
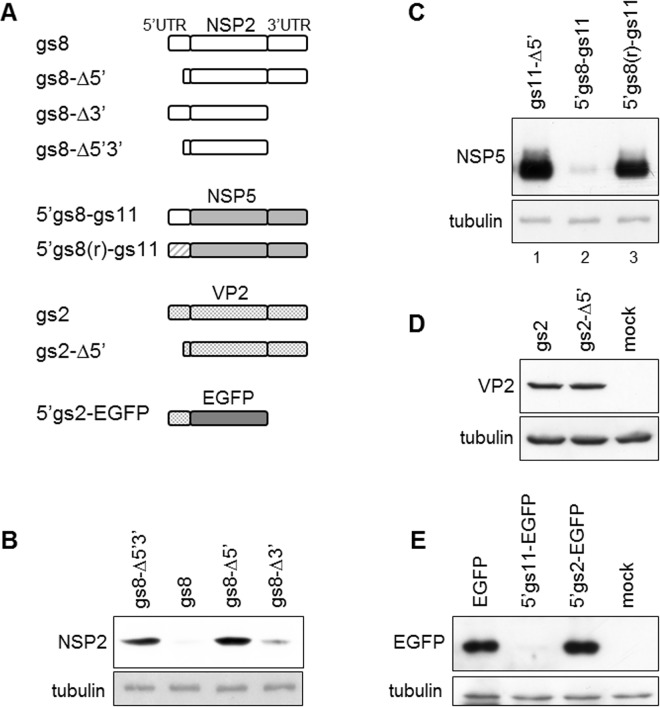
The 5’UTRs of gs2 and gs8. A) Scheme of the constructs used. B-E) WB analysis with the indicated antibodies of MA104 cells transfected with: gs8 constructs with and without 5’ and 3’ UTRs (B); chimeric constructs with 5’UTR from gs8 and NSP5 ORF (C); gs2 constructs with or without 5’UTR (D); constructs with EGFP ORF downstream of the 5’UTR of either gs11 or gs2 (E). Tubulin used as loading control.

We next tested whether the 5’UTRs of two other rotavirus genome segments shared the same activity as the gs11 5’UTR. The 46-nucleotide long 5’UTR of gs8 that encodes the nonstructural protein NSP2 was able to strongly downregulate expression of NSP2 (Fig 2B) as well as of NSP5 encoded by a chimeric construct with the gs8 5’UTR replacing the 5’UTR of gs11 (5’gs8-gs11) (Fig 2C). A chimeric construct containing a reshuffled nucleotide sequence version of the gs8 5’UTR [5’gs8(r)-gs11] was not inhibitory (Fig 2C, lane 3) suggesting that the inhibitory activity was dependent on the 5’UTR primary sequence.

In contrast to gs8 and gs11, the 14-nucleotide long 5’UTR of gs2 that encodes the structural protein VP2 did not inhibit protein expression with the ORF of either the full-length viral protein VP2 (Fig 2D) or EGFP (Fig 2E).

### Inhibition depends on 5’UTR primary sequence

**Fig 3:**
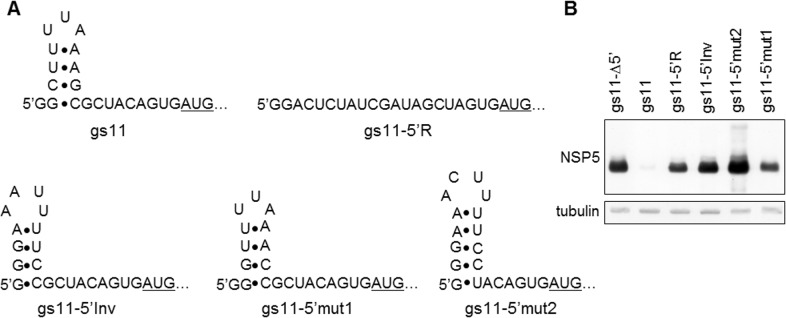
Inhibition depends on 5’UTR primary sequence. A) Predicted structures of gs11 5’UTR and mutants. B) Anti-NSP5 WB of MA104 cells transfected with the different mutant constructs shown in A; gs11-Δ5’ was included as a positive control. Tubulin used as loading control.

The full-length gs11 5’UTR is 21-nucleotide long and for this region the RNAfold software predicts a stem-loop structure (shown in Fig 3A). We thus addressed whether gs11 downregulation was dependent on the secondary structure or on the primary sequence of the 5’UTR. Four different gs11 constructs with the 5’UTR modified in different ways were engineered (Fig 3A). In one case the sequence was reshuffled preserving the first two guanines at the 5’ end (to allow T7 transcription initiation and preserve 5’ end sequence) and the last three nucleotides immediately upstream of the translation start codon AUG (construct gs11-5’R). This construct is predicted not to form the stem-loop structure. In a second construct the stem was preserved, inverting the nucleotides on each arm (construct gs11-5’Inv). A third one contained two complementary mutations (C3G and G11C) at the base of the stem that should also preserve a stem-loop structure (construct gs11-5’mut1). A final construct (gs11-5’mut2) contained a sequence that should also maintain a similar stem-loop structure. When tested in MA104 cells, only the wt 5’UTR showed strong protein expression impairment (Fig 3B) indicating that the inhibitory activity was the consequence of a primary sequence contained in the gs11 5’UTR, hereafter referred to as inhibitory motif (IM).

### A 6-nucleotide long motif on 5’UTR

**Fig 4:**
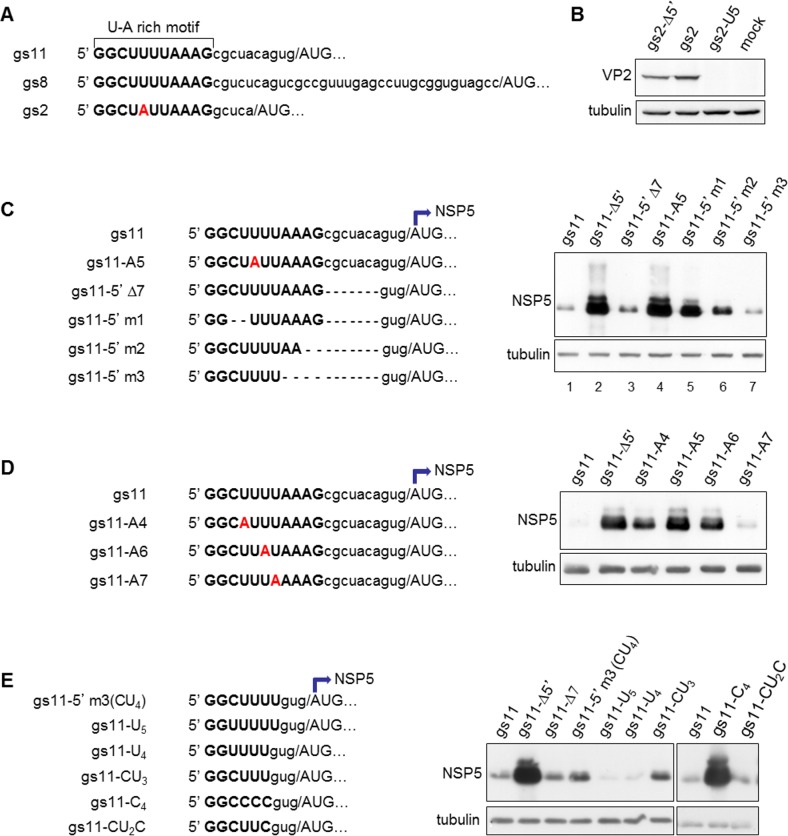
A 6-nucleotide long motif on 5’UTR. A) Alignment of 5’UTRs from gs11, gs8 and gs2. The 11-nucleotide long U-A rich motif is highlighted (bold). The different nucleotide in position 5 in gs2 is shown in red. B) Anti-VP2 WB of MA104 cells transfected with the indicated gs2 constructs. C) Right panel: anti-NSP5 WB of cells transfected with the mutant constructs shown on left panel. D) Anti-NSP5 WB of cells transfected with the indicated gs11 U-to-A mutants shown on left panel. E) Anti-NSP5 WB of cells transfected with constructs containing the 5’ terminal nucleotides of the U-A rich motif and with mutants of the pyrimidine tract. Tubulin used as loading control.

Alignment of the 5’UTRs of gs2, gs8, and gs11 showed homology within the initial 5’ ends of all three sequences (Fig 4A). An 11-nucleotide long U-A rich motif is conserved between gs8 and gs11, while in gs2 there is a single difference (A5 instead of U5). Since gs2 5’UTR was not inhibitory (Fig 2D–2E), we tested a construct with an A5U mutation introduced in 5’UTR of gs2 cDNA (gs2-U5), making this part identical to the motif conserved in gs8 and gs11. As shown in Fig 4B, the A5U mutation had a strong inhibitory effect on VP2 expression.

In order to map IM, additional mutants on gs11 5’UTR were engineered (Fig 4C, left panel). An U5A mutation was introduced in the 5’UTR of gs11 to make it identical to gs2 (gs11-A5); in a second mutant the seven nucleotides downstream of the U-A rich motif and up to the last three nucleotides upstream of the AUG start codon (from C12 to A18) were deleted (construct gs11-5’Δ7). When tested in MA104 cells, the U5A mutation completely abolished inhibition, while gs11-5’Δ7 showed strong inhibitory activity (Fig 4C, lanes 1–4). Further mapping was carried out by deleting nucleotides within the U-A rich motif on the gs11-5’Δ7 mutant (schemes in Fig 4C). While deletion of the last two or four nucleotides (AG in gs11-5’m2; AAAG in gs11-5’m3, respectively) still showed expression inhibition (Fig 4C, lanes 6–7), deletion of the two nucleotides immediately downstream of the initial dinucleotide GG (CU, gs11-5’m1) allowed high expression levels (Fig 4C, lane 5). Thus, it appears that nucleotides in positions 3, 4, and 5 are essential components of IM. Indeed, comparative analyses of single U to A mutations in positions 4 to 7 built on the wild-type gs11 background (constructs gs11-A4, gs11-A5, g11A6 and gs11-A7) showed that U in positions 4, 5 and 6 are the relevant ones, although the U-to-A mutation in position 4 determined a lower rescue of protein expression as compared to the same mutations in positions 5 and 6 (Fig 4D). A new set of mutants was then built on the gs11-5’m3 inhibitory construct, which has the terminal 7 nucleotides of the U-A rich motif initially identified. As shown in Fig 4E, mutation of C3 into U (GGUUUUU, gs11-U_5_), or deletion of C3 (GGUUUU, gs11-U_4_) showed even stronger inhibition than the parental construct containing CU_4_, while construct gs11-CU_3_ (GGCUUU) was as inhibitory as the parental one. Further mutations were performed on gs11 mutating U4, U5 and U6 into C (GGCCCC, gs11-C_4_) or the single U6 into C (GGCUUC, gs11-CU_2_C). While the single U6C mutation did not show any effect, mutation of also U4 and U5 completely restored protein expression. Thus, downstream of the 5’ terminal GG dinucleotide present in all rotavirus mRNAs, IM requires at least one pyrimidine in position 3 followed by two uracils and a pyrimidine in position 6.

### IM 5’ terminal position is essential

**Fig 5:**
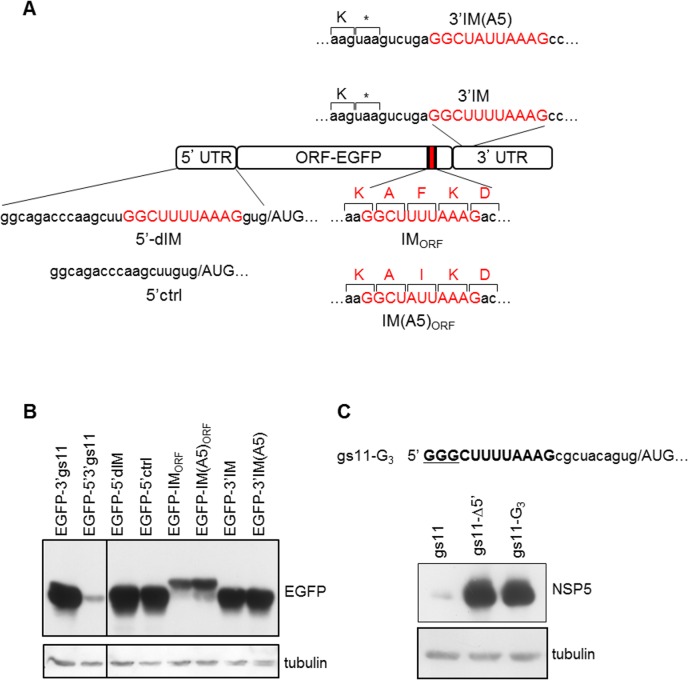
IM 5’ terminal position is essential. A) Schematic representation of constructs with the 11-nucleotide long U-A rich motif (containing IM) positioned within gs11 5’UTR (construct 5’-dIM), in EGFP ORF (construct IM_ORF_) or in gs11 3’UTR (construct 3’IM). Within the 5’UTR the motif was placed in a non 5’-terminal position, downstream of a 15-nucleotide long not inhibitory sequence; as a control, a construct with the same sequence without the U-A rich motif was used (5’ctrl). In the construct with the motif within the ORF (IM_ORF_), the amino acids encoded by the inserted motif and by the U5A control mutant [IM(A5)_ORF_] are shown. Within the 3’UTR, the motif or the U5A control mutant [3’IM(A5)] were positioned six nucleotides downstream of the stop-codon. B) Anti-EGFP WB of extracts from cells transfected with the indicated constructs. The EGFP construct with only gs11 3’UTR was used as a negative control while construct EGFP-5’3’gs11 was used as a positive control. C) Anti-NSP5 WB of cells transfected with a gs11 mutant construct with an additional 5’-terminal G (gs11-G3). Tubulin used as loading control.

We next examined whether the position of IM within the mRNA is of any relevance for its inhibitory activity. Thus, the originally identified 11-nucleotide long U-A rich motif (GGCUUUUAAAG) of gs11 was moved from its original 5’ end terminal position to a position either within the 5’UTR, downstream of a 15-nucleotide long arbitrary sequence that does not inhibit protein expression, or in the coding region or in the 3’UTR of a construct containing the EGFP ORF and the UTRs of gs11. The resulting constructs, indicated as 5’-dIM, IM_ORF_ and 3’IM, respectively, are schematically shown in Fig 5A. As controls, the 15-nucleotide long 5’UTR without IM (5’ctrl) and IMs with the U5A mutation [IM(A5)_ORF_, 3’IM(A5)] were used. Expression downregulation was only present when IM was positioned at the 5’-terminal end in the 5’UTR, with GG as the initial dinucleotide, while insertion of the wild-type IM or the U5A control within the ORF or the 3’UTR did not show any difference (Fig 5B). This conclusion was further supported with a single insertion mutant with one additional 5’-terminal G added in gs11 (gs11-G_3_), which showed strong expression levels (Fig 5C) thus indicating that IM strictly requires a 5’ terminal position to downregulate expression. Therefore, IM can be defined as a 5’ terminal sequence containing two guanines (found in all mRNAs of rotaviruses of all groups) followed by a pyrimidine in position 3, and, at least in gs11, three uracils in positions 4–6.

### Compromised expression is due to both T7-mediated transcription and cellular translation

**Fig 6:**
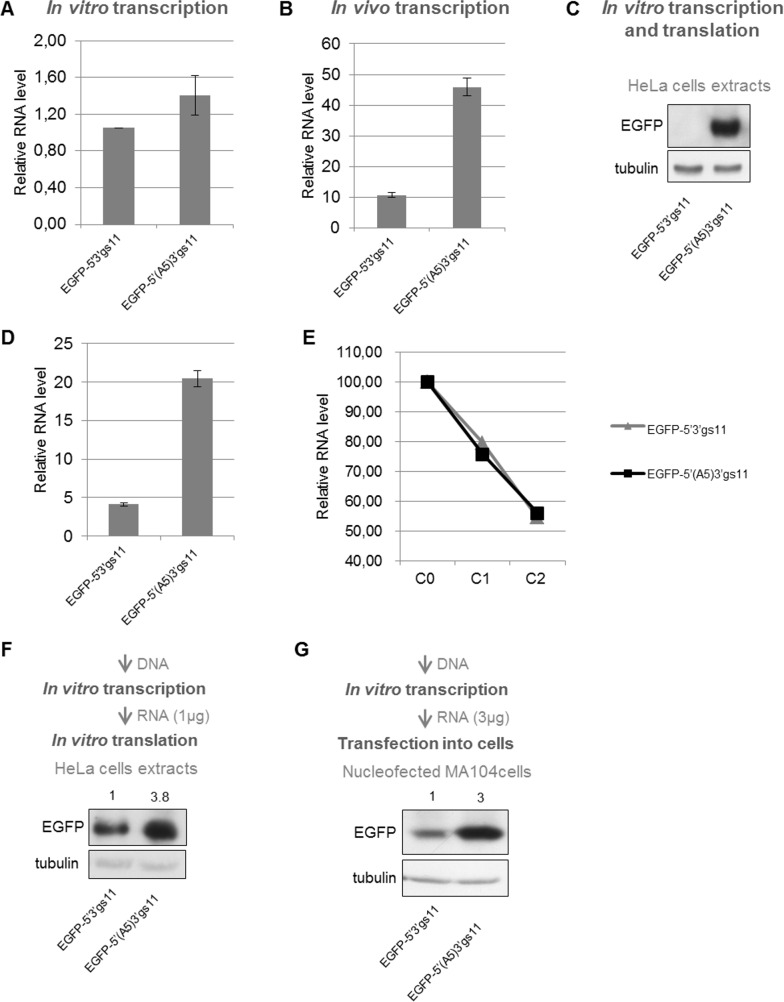
Compromised expression is due to T7-mediated transcription and translation. Yield of transcripts and protein products of constructs containing EGFP ORF flanked by gs11 UTRs in wild-type and the U5A version. A) Comparison of mRNAs yield after *in vitro* transcription with T7 polymerase in transcription buffer. B) qPCR of the same constructs transfected in MA104 cells. C) WB of EGFP protein produced following *in vitro* coupled transcription/translation in a HeLa cell lysate-based kit. D) mRNA yields (by qPCR) from the same *in vitro* coupled transcription/translation HeLa cell lysate-based kit. E) Decay rate of transcripts in MA104 cells, quantified by qPCR. F) Anti-EGFP WB of samples obtained following *in vitro* translation (in the HeLa cell lysate-based kit) of mRNAs pre-synthesized as in A. G) Anti-EGFP WB of MA104 cells electroporated with mRNAs pre-synthesized as in A. Tubulin used as loading control.

We then determined whether compromised expression was the consequence of inhibition of transcription or translation. mRNAs were produced *in vitro* by T7 polymerase from a construct containing the EGFP ORF flanked by gs11 UTRs (EGFP-5’3’gs11) and from the corresponding one containing the U to A mutation in position 5 [EGFP-5’(A5)3’gs11]. Both mRNAs were produced at similar levels, with the last one transcribed only 1.5 times more (Fig 6A). Transcription *in vivo* from the same two constructs in MA104 cells infected with vTF7.3 vaccinia virus (determined by qPCR) yielded, however, a five times higher level of the transcript from the U5A construct (Fig 6B). When tested in a transcription/translation *in vitro* system derived from HeLa cells, qPCR analysis revealed a difference in the relative amount of mRNAs comparable to the one detected *in vivo* (Fig 6D), while the Western blot consistently confirmed reduced protein expression at a similar extent (Fig 6C). Altogether these data indicate that IM plays a strong effect on T7 transcription only in presence of cytoplasmic components (cells or *in vitro* systems based on cellular extracts).

To establish if IM has also an impact on transcripts stability, an assay of the decay rate was performed exploiting incorporation of the ribonucleotide homologue ethynyl uridine (EU), in presence of pactamycin and cycloheximide (two drugs that block translation). EU was fed to transfected cells for 1.5 hours and then incorporation into newly-made mRNAs was stopped by addition of the transcription inhibitor actinomycin D. RNA samples were collected at different time points and EU-labelled mRNAs quantified by qPCR. As shown in Fig 6E, there was no difference in the relative decay rate of the two types of transcripts. Therefore, while having an impact on T7-mediated transcription, IM does not affect transcripts stability.

We next evaluated whether IM has an effect on translation. The EGFP-5’3’gs11 construct and the EGFP-5’(A5)3’gs11 mutant were *in vitro* transcribed and then equal amounts of transcripts were *in vitro* translated in a HeLa cell lysate-based protein expression system. As shown in Fig 6F, the transcript with IM mutated in position 5 was translated 3.8 times more. The difference in the EGFP levels, however, was not as much as that observed when administering the two cDNAs to the same *in vitro* (coupled transcription/translation) expression system (compare Fig 6F with 6C). In order to confirm impaired translation also *in vivo*, equal amounts of *in vitro* transcribed mRNAs were electroporated into MA104 cells. The three times difference observed was comparable to that obtained *in vitro* (Fig 6G). Of note, to obtain detectable levels of proteins, the mRNAs transfected contained polyA tails that did not affect IM activity (S1 Fig). Because of the controversial role of the non-structural protein NSP3 in regulating translation of rotavirus mRNAs [26,27], we tested whether NSP3 was able to increase expression of NSP5 from constructs with the full-length 5’UTR. NSP3 was overexpressed (from constructs without UTRs) in two different versions, un-tagged or SV5 N-terminally tagged. NSP3 did not revert the inhibitory effect of the 5’UTR on NSP5 expression and did not affect either NSP5 expression from mRNAs with the 5’UTR deleted (S2 Fig).

In conclusion, the overall IM inhibitory effect on expression is due to an interference with both T7-mediated transcription and cellular translation.

### The UTRs of SA11 strain genome segments

**Fig 7:**
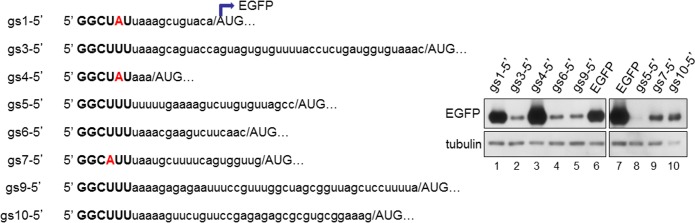
The UTRs of SA11 strain genome segments. Anti-EGFP WB of cells transfected with constructs containing the EGFP ORF fused to the 5’UTR of the different gs indicated on the left. Tubulin used as loading control.

We tested the 5’UTRs of all remaining SA11 genome segments cloned upstream of EGFP. As expected, the 5’ UTRs of gs 3, 5, 6, 9, and 10 (encoding VP3, NSP1, VP6, VP7 and NSP4, respectively), which contain an IM identical to that of gs11, inhibited EGFP expression, whilst those of gs 1 and 4 (encoding VP1 and VP4, respectively) containing an A in position 5 did not (Fig 7). Interestingly, the 5’UTR of gs7 (encoding NSP3) that contains an A in position 4 also affected EGFP expression (Fig 7, lane 9), suggesting that the nucleotide in position 4 may be either adenine or uracil. Therefore a more precise definition of IM corresponds to a 5’ terminal sequence containing two guanines followed by a pyrimidine in position 3, uracil or adenine in position 4 an uracil in position 5 and a pyrimidine in position 6, 5’-GGY(U/A)UY-3’.

Thus, a set of cDNA constructs encoding all genome segments under the control of T7 promoter and containing either of the described mutations (addition of an extra 5’ G or mutation of T5 into A) with exception of gs1, 2 and 4 should provide the basis for a more efficient helper virus-free reverse genetics system.

### Virus infection overcomes IM activity

**Fig 8:**
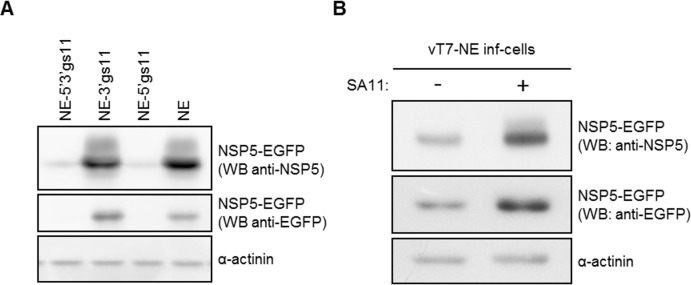
Rotavirus partially reverts IM-mediated expression inhibition. A) Anti-EGFP and anti-NSP5 WB of MA104 cells transfected with constructs containing the NSP5-EGFP (NE) ORF with or without gs11 5’ and 3’ UTRs, as indicated. B) Anti-EGFP and anti-NSP5 WB of MA104 cells infected with SA11 and the recombinant vaccinia virus vT7-NE expressing NSP5-EGFP from an mRNA with gs11 5’ and 3’ UTRs. α-actinin as loading control.

To test whether rotavirus infection can overcome IM activity, we constructed a recombinant vaccinia virus (vT7-NE) with inducible expression of both the T7 RNA polymerase and an NSP5-EGFP fusion (NE) flanked by the gs11 UTRs. This construct was first validated by transfection experiments showing that the gs11 5’UTR inhibited NSP5-EGFP expression, independently of the 3’UTR (Fig 8A). Co-infection of MA104 cells with both vT7-NE and SA11 was close to 100%, according to immunofluorescence with anti-EGFP and anti-NSP4 antibodies (not shown). As shown in Fig 8B, rotavirus infection partially reverted the inhibitory effect of the gs11 5’UTR on NSP5-EGFP, indicating that either a viral factor or a virus-induced cellular factor(s) allows high-level expression of mRNAs containing IM in their 5’UTR.

## Discussion

Knowledge of the function of several rotavirus proteins and their precise roles in the viral replication cycle still presents unclear points. In part this is due to the lack of a broad reverse genetics system that could be universally applicable to all RV genome segments. Plasmid-based helper virus-free strategies were already successful with other viruses belonging to the same family of rotavirus (orbivirus and orthoreovirus) but failed when applied to rotavirus. One of the main reasons proposed to explain the difficulties encountered in having a helper virus-free system for rotavirus is the poor expression of transfected mRNAs or cDNAs. Indeed in the few cases in which recombinant RVs were obtained, robust protein production was guaranteed by the helper virus infection.

The experience with bluetongue virus and the “double transfection strategy” highlighted the importance of a strong protein production in order to achieve the recovery of recombinant virus [28]. It is indeed believed that the first round of transfection supplies the proteins necessary for the formation of replication complexes, which then act on the transcripts introduced during the second transfection.

In this work we revealed that the 5’UTR of several (but not all) genome segments of rotavirus contain an inhibitory motif (IM) that downregulates expression of rotavirus cDNAs when expressed exploiting a T7 polymerase-encoding recombinant vaccinia virus. This is the preferred system used in all attempts to generate a recombinant rotavirus since the T7 promoter coupled with the HDV ribozyme provides *bona fide* 5’ and 3’ terminal sequences of viral mRNAs.

Rotavirus mRNAs are known to form secondary structures involving interaction between 5’ and 3’ UTRs [7,29], but IM activity proved to be independent of the ORF and the 3’UTR sequence; indeed, the simple addition of an IM-containing 5’UTR upstream any ORF sequence was sufficient to inhibit protein expression. The effect was tested on the ORFs of several viral proteins (NSP5, NSP2, VP2) and of EGFP, used as a reporter protein. Furthermore, IM activity is dependent on the primary sequence and not related to secondary structures in 5’UTR, as proved by constructs gs11-5’R, gs11-5’Inv, gs11-5’mut1, gs11-5’mut2 (Fig 3), but also by construct gs11-5’mut3 (Fig 4C), which maintains the IM sequence but whose secondary structure has been disrupted by deletion of eleven nucleotides. The IM sequence that we have identified includes at least four nucleotides after the initial GG dinucleotide present in all genome segments of all rotavirus groups. Since the addition of a single G at the 5’ end was sufficient to abolish IM inhibitory effect, we defined IM as the 5’ terminal hexanucleotide 5’-GGY(U/A)UY-3’. Interestingly, the 5’ terminal ends of genome segments of orthoreovirus and bluetongue virus do not have sequence homology with rotavirus IM since their conserved sequences are 5’-GCUA-3’ and 5’-GUUAAA3’, respectively. Besides, a single U to A mutation in position 5 of gs11 5’UTR was enough to make IM no longer functional and A5 is naturally present in gs1, gs2 and gs4 of strain SA11, whose 5’UTRs indeed did not downregulate protein expression. Exploiting EGFP as a reporter protein, we instead confirmed inhibitory activity of the 5’UTRs of the remaining eight SA11 rotavirus genome segments that contain IM (gs3, 5, 6, 7, 8, 9, 10 and 11).

According to our studies, IM activity is the consequence of impairment of both transcription and translation. Quantification of the relative levels of mRNAs showed that *in vivo* there is a higher rate of transcription of the cDNAs in which IM was mutated in position 5. T7 polymerase is a single subunit enzyme that does not require protein co-factors. The T7 promoter is composed of two domains: the binding region and the catalytic domain. Since the binding region is located upstream position -5, IM is unlikely capable of interfering with the proper tight binding of the polymerase with its promoter [30]. During the first phase of transcription, T7 polymerase produces short transcripts of 2–6 nucleotides and only after the synthesis of 10/12-nucleotide long RNAs, it enters the elongation phase that stabilizes the polymerase-DNA complex [31]. In this step, T7 polymerase rearranges creating a channel that accommodates a 7-nucleotide long hetero-duplex, and a tunnel for the exit of transcripts [32]. The IM nucleotide sequence could interfere with the processivity of transcription and T7 polymerase could therefore indulge in abortive falloff after incorporation of U, while sequences with either three 5’ terminal G or an A in position 5 might help T7 polymerase in the transition from the initiation to the elongation phase [33].

Interestingly, the quantification of mRNA levels using two different *in vitro* transcription kits presented a discrepancy. The mRNA levels in the coupled transcription-translation HeLa cell lysate-based system reflected what was observed *in vivo* (also for the level of expressed protein), while in the *in vitro* transcription kits containing only T7 enzyme and an adequate transcription buffer, transcripts of cDNAs containing non-functional IM were only slightly more abundant than the counterpart. We questioned if this yield disparity could be explained by a major rate of degradation that could occur *in vivo* to IM-containing mRNAs. Our results showed no differences in mRNA stability. Since the difference in transcription levels was detectable only in presence of cellular cytoplasmic extracts, a possible explanation hypothesizes the presence of a cytoplasmic factor that can specifically bind to IM and therefore interfere with transcription.

The presence of IM affects also translation since mRNAs containing non-functional IM increase protein expression by 3 times compared with the ones containing functional IM. This difference was observed both *in vivo*, transfecting *in vitro* synthesized transcripts into cells, and *in vitro*, using HeLa cell lysate-based *in vitro* protein expression systems fed with pre-formed mRNAs. This effect on translation upon deletion of the 5’UTR from gs11 was not due to a change of the nucleotide context of the start codon. Optimization of the nucleotides upstream the AUG codon did not modify expression of gs11 nor of the mutant lacking the 5’UTR. In both constructs, the nucleotide in position +4, which is not optimal in gs11 (U instead of G), was not changed. In a recent work with gs4 Gratia et al. reported that optimisation of both positions -3 and +4 is important for expression efficiency [34]. However, the strong difference in expression of the two constructs with start codons sharing the same nucleotide context (GUG**AUG**U), strongly suggests that the IM inhibitory activity on translation cannot be ascribed to a less effective start codon recognition. The UTRs of a number of viruses, such as JEV [35], HIV-1 [36], HCV [37–40], and poliovirus [41] are bound by cellular proteins (such as protein La) with an effect of translational enhancement. This binding occurs via a polypyrimidine tract inserted in the context of specific secondary structures (i.e. IRES) in close proximity to the AUG codon. Our data show that the 5’UTR secondary structure of rotavirus mRNAs does not affect translation efficiency. Interestingly, however, those cellular proteins that affect viral protein translation of the above mentioned viruses also regulate translation of a class of cellular mRNAs containing a motif similar to IM, the so-called TOP mRNAs (TOP = Terminal OligoPyrimidine) [42]. These mRNAs encode various components of the translational machinery (such as some ribosomal proteins and elongation factors) and contain a rather short 5’UTR that starts with a C followed by an uninterrupted tract of 4–14 pyrimidines, generally located upstream of a CG-rich sequence (reviewed in [42]). Although the molecular mechanisms regulating TOP mRNA translation are still poorly defined, the TOP motif is recognized by specific trans-acting factors, some with positive and others with negative effects [43–45]. A common feature of the TOP motif and IM is the requirement of the 5’ end terminal position [46]. In fact, we observed that IM loses its translation downregulation activity when moved from the 5’ end terminal position, suggesting a regulatory role at the initiation step of translation.

Recently, it has been reported that certain adenosines within the 5’UTRs of cellular mRNAs are preferentially methylated under stress conditions and this post-transcriptional modification was found to promote translation initiation [47]. Although of different nature, the presence of A instead of U in position 5 of some viral 5’UTRs might represent a factor that favors translation.

We found that rotavirus infection partially reverts IM-mediated expression inhibition suggesting that a viral component (or a cellular factor induced upon infection) interferes with the inhibitory mechanism in uninfected cells. It can be hypothesized that while in uninfected cells a trans-acting negative regulator has free access to the viral mRNAs containing an IM, in infected cells it is displaced from viral messengers possibly through binding competition with specific viral proteins. In this respect, we performed co-expression experiments with a panel of viral proteins (VP1, VP2, VP3, VP6, NSP2, NSP4, NSP5). So far, we have not identified any individual viral protein able to revert IM inhibitory activity. Not even NSP3 could rescue protein expression. In the context of viral infection, specific changes in subcellular distribution and in quantity of the translation apparatus components, [48–50] result in a complex interaction network between multiple viral and cellular factors mediating efficient translation of all viral mRNAs. In fact, during rotavirus infection most cellular mRNAs are confined to the nucleus, where become hyperadenylated and unable to reach the cytoplasm [48]. In addition, several signalling pathways are activated that can affect the cellular translation apparatus. Overexpression of a single viral protein appears not to be sufficient to revert expression.

IM and its non-functional versions are highly conserved among the same genome segments of different group A rotavirus strains (S4 Table). The presence of the inhibitory motif in 8 out of the 11 genome segments, with the consequent low level of viral mRNAs and proteins, can easily explain why transcripts of rotavirus cDNAs are not infectious. Addition of support plasmids engineered to enhance RV protein expression has already been proposed [51]. Our data suggest the possibility of reaching the same goal with either of two single base mutations (addition of a third G upstream 5’UTR/T5A mutation) in order to generate RV cDNAs that could sustain a more robust production of both mRNAs and proteins. These plasmids can be employed in the attempt to recover recombinant rotavirus upon transfection of the eleven cDNAs, reproducing the strategy that was already successful with mammalian orthoreovirus. Importantly, incorporation into an infectious viral particle of a genome segment with an additional or two additional G upstream 5’UTR of gs 8 was already observed [14].

In conclusion, our study is a deep analysis of the mechanisms underlying poor protein expression from rotavirus cDNAs and considers their possible involvement in the failure of helper virus-free reverse genetics strategies applied to rotavirus. We mapped two distinct single base mutations that increase both mRNA transcription and protein translation, which are likely compatible with genome segment packaging in infectious particles. The increased yields of mRNAs and proteins could be the key to obtaining infectious virus from cells transfected with all eleven genome segments.

## Supporting Information

S1 Fig The poly-A tail does not affect downregulation by gs11 5’UTR. Anti-NSP5 WB of cells transfected with polyadenylated versions of gs11 constructs, with or without 5’UTR. The polyadenylated mRNAs were T7-polymerase transcribed, derived from a synthetic gene fragment containing 30 adenines downstream of the 3’UTR. Tubulin used as loading control.
(TIF)

S2 Fig NSP3 does not affect downregulation by gs11 5’UTR. A-B) Anti-NSP5 WB of MA104 cells co-expressing different gs11 constructs with or without NSP3, either un-tagged (A) or SV5-tagged (B). In B anti-SV5 WB revealing expression of SV5-NSP3. Tubulin used as loading control.
(TIF)

S1 Table Primers used to construct plasmids containing rotavirus genome segments, either full-length or lacking one or both UTRs. (XLS)

S2 Table Primers used for site-specific mutagenesis. (XLS)

S3 Table Oligonucleotides for construction of plasmids containing the 5’UTRs of the indicated genome segment upstream the EGFP ORF. (XLS)

S4 Table IM in genome segments encoding structural and non-structural proteins of five different tissue culture-adapted (simian SA11, porcine OSU, simian RRV, human Wa, human DS-1) and one circulating human wild-type (RVA/Human-wt/BGD/Dhaka16/2003/G1P[8]) group A rotavirus strains, shown in bold (GenBank accession numbers: SA11, NC_011500-NC_011510; OSU, KJ45084-KJ45094; RRV, EU636924-EU636934; Wa, JX406747-JX406757; DS-1, HQ650116-HQ650126; wt RVA, DQ492669-DQ492679). Nucleotides that render IM non-functional are indicated in red; genome segments containing a functional IM are marked with a tick. ALL indicates the six strains.
(TIF)
