# Supplementary figures and images for: An Inhibitory Motif on the 5’UTR of Several Rotavirus Genome Segments Affects Protein Expression and Reverse Genetics Strategies
Source: PLoS One. 2016 Nov 15;11(11):e0166719. doi: 10.1371/journal.pone.0166719 (PMC5112996; doi:10.1371/journal.pone.0166719)

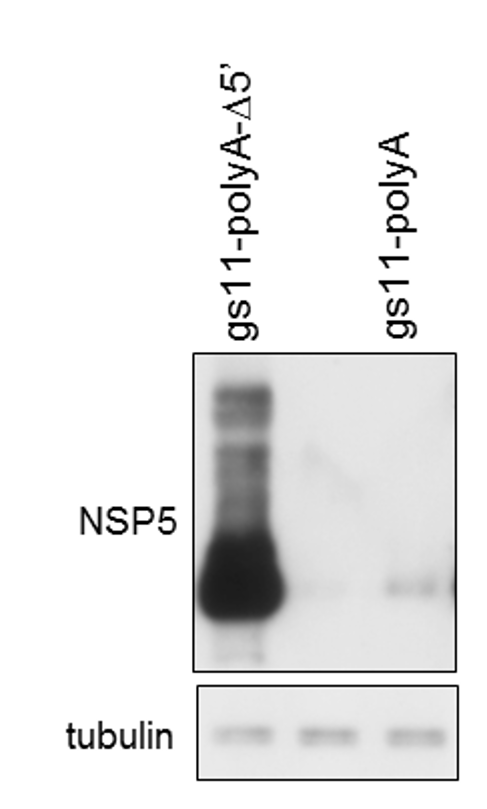

Supplement: S1 Fig — Anti-NSP5 WB of cells transfected with polyadenylated versions of gs11 constructs, with or without 5’UTR. The polyadenylated mRNAs were T7-polymerase transcribed, derived from a synthetic gene fragment containing 30 adenines downstream of the 3’UTR. Tubulin used as loading control. (TIF) [file pone.0166719.s001.tif]

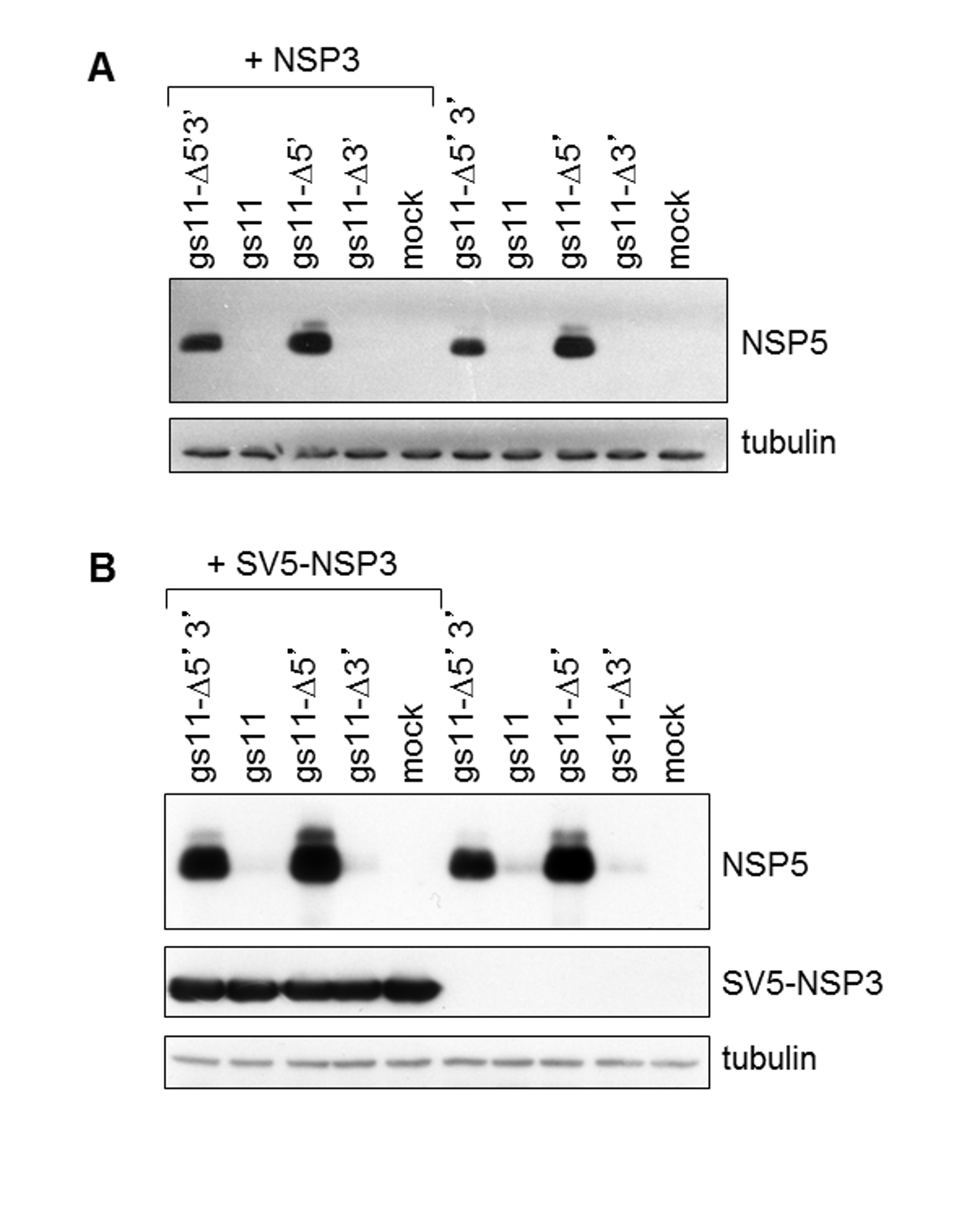

Supplement: S2 Fig — A-B) Anti-NSP5 WB of MA104 cells co-expressing different gs11 constructs with or without NSP3, either un-tagged (A) or SV5-tagged (B). In B anti-SV5 WB revealing expression of SV5-NSP3. Tubulin used as loading control. (TIF) [file pone.0166719.s002.tif]

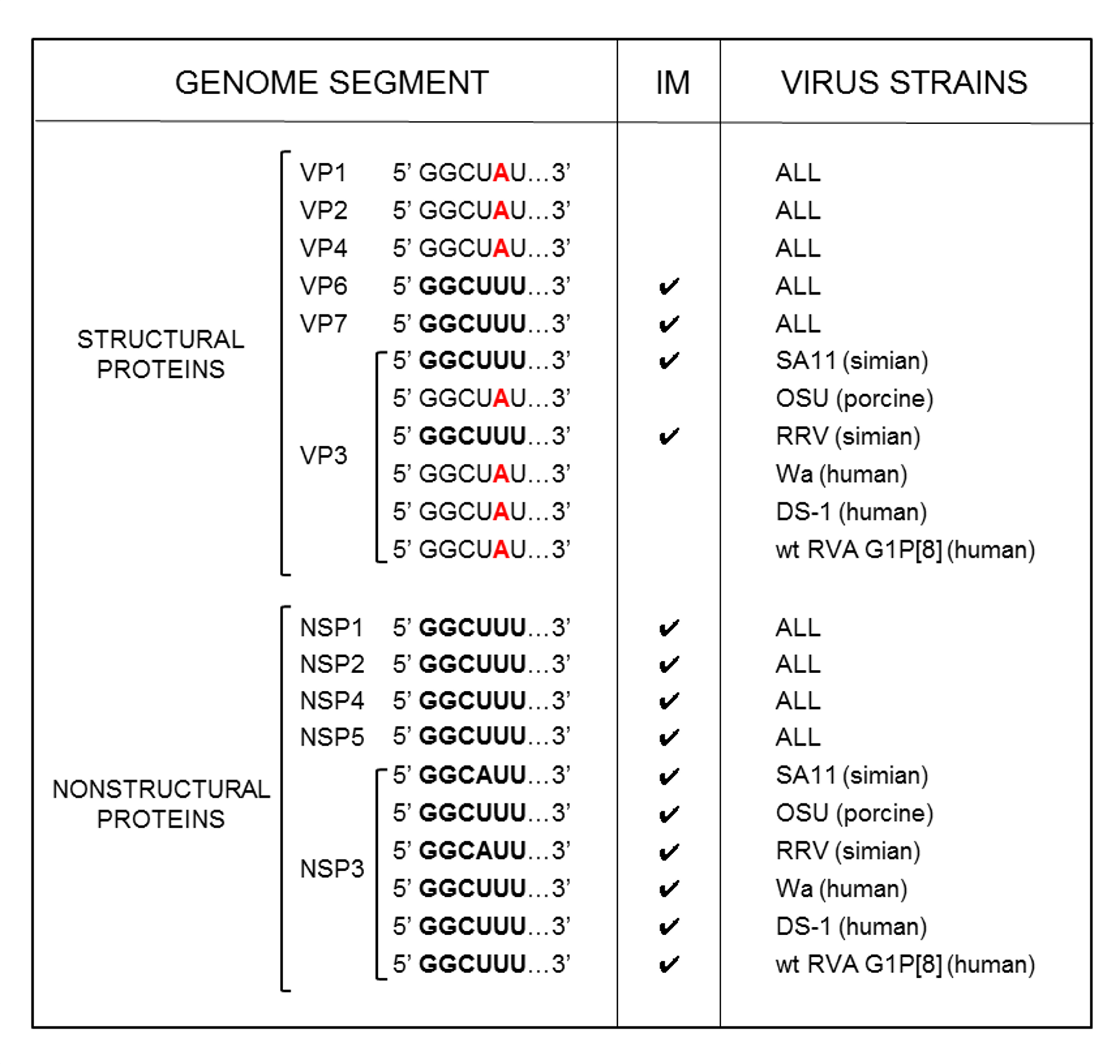

Supplement: S4 Table — Nucleotides that render IM non-functional are indicated in red; genome segments containing a functional IM are marked with a tick. ALL indicates the six strains. (TIF) [file pone.0166719.s006.tif]
